# Mechanochemical Applications of Reactive Extrusion from Organic Synthesis to Catalytic and Active Materials

**DOI:** 10.3390/molecules27020449

**Published:** 2022-01-10

**Authors:** Emanuela Calcio Gaudino, Giorgio Grillo, Maela Manzoli, Silvia Tabasso, Simone Maccagnan, Giancarlo Cravotto

**Affiliations:** 1 Department of Drug Science and Technology, University of Turin, Via P. Giuria 9, 10125 Turin, Italy; emanuela.calcio@unito.it (E.C.G.); giorgio.grillo@unito.it (G.G.); maela.manzoli@unito.it (M.M.); 2 Department of Chemistry, University of Turin, Via P. Giuria 7, 10125 Turin, Italy; silvia.tabasso@unito.it; 3 Gimac International Srl, Via Roma 5/7, 21040 Castronno, Italy; simone.maccagnan@gimac.com; 4 World-Class Research Center, Sechenov First Moscow State Medical University, 8 Trubetskaya ul, 119991 Moscow, Russia

**Keywords:** solvent free, process intensification, reactive extruder, solid-phase flow chemistry, green chemistry

## Abstract

In the past, the use of mechanochemical methods in organic synthesis was reported as somewhat of a curiosity. However, perceptions have changed over the last two decades, and this technology is now being appreciated as a greener and more efficient synthetic method. The qualified “offer” of ball mills that make use of different set-ups, materials, and dimensions has allowed this technology to mature. Nevertheless, the intrinsic batch nature of mechanochemical methods hinders industrial scale-ups. New studies have found, in reactive extrusion, a powerful technique with which to activate chemical reactions with mechanical forces in a continuous flow. This new environmentally friendly mechanochemical synthetic method may be able to miniaturize production plants with outstanding process intensifications by removing organic solvents and working in a flow mode. Compared to conventional processes, reactive extrusions display high simplicity, safety, and cleanliness, which can be exploited in a variety of applications. This paper presents perspective examples in the better-known areas of reactive extrusions, including oxidation reactions, polymer processing, and biomass conversion. This work should stimulate further developments, as it highlights the versatility of reactive extrusion and the huge potential of solid-phase flow chemistry.

## 1. Introduction

Over the last few decades, mechanochemistry has been widely explored as a powerful tool with which to perform environmentally benign and sustainable chemical syntheses, as mechanical energy, in the form of compression, shear, and friction, is able to boost chemical transformations [1,2]. Research in this area has steadily increased from 1985, driven by the advantages provided by mechanochemistry. Moreover, this technique has not only proven to be a viable alternative to traditional solution-based syntheses, but it has also gained even greater attention thanks to its ability to allow reactions to take new reactive paths, thus “unlocking” novel compounds that would be inaccessible via conventional synthetic methods [3,4,5,6]. Many additional advantages, including reaction telescoping, higher yields, and selectivities, together with greater safety, have been reported for mechanochemical reactions [7,8,9,10,11]. The increasing demand for cleaner processes and eco-conscious reaction conditions means that mechanochemistry has now found great use in many applications, not only in organic [12] and organometallic syntheses [3], but also in metal [9,10,13] and enzymatic catalysis [14], in co-crystal formulations [15], metal-organic framework (MOF) preparations [16], and in the synthesis of active pharmaceutical materials and ingredients (APIs) [17]. This has recently resulted in a direct acknowledgement of mechanochemistry, as one of the “top ten emerging technologies in chemistry” by the International Union of Pure and Applied Chemistry (IUPAC) [18]. However, the scalability of this solvent-free chemical technology towards commercial manufacturing is currently a challenge.

Historically [2], the first mechanochemical reactions were performed by grinding reactants together in a mortar with a pestle, an approach that is sometimes referred to as “grindstone chemistry”. While this technique does not require specialized equipment and is therefore feasible in any laboratory, it suffers from some limitations. Indeed, it requires short reaction times if it is to be viable. Furthermore, reproducibility is sometimes challenging, as it is dependent on the physical strength of the operator. Automated ball mills have more recently been introduced for laboratory-scale syntheses. These instruments allow the energy input to be controlled by adjusting the milling frequency and, therefore, show better reproducibility. In addition, they are safer, as the reactions take place in closed vessels and the operator is not exposed to the reactants, catalysts, and products. Research on the scale-up of these mechanochemical reactions is also ongoing and focuses primarily on the use of larger planetary ball mills [19,20,21]. It is worth noting that extrusion techniques have very recently begun to be explored as remarkably effective alternatives to milling that can circumvent these problems and move to continuous, scalable mechanochemical synthesis. The term extrusion refers to a family of continuous processing techniques, in which materials are forced through constrained spaces, which involves the intense mixing of materials [22], and screw extruders (SE) have recently been included in the family of mechanochemistry tools [23]. In this instrumental setup, solid reagents are ground together by a rotatory screw while the mixture is transported along the extrusion path through a barrel. Different sections or zones of the screw can be readily exchanged to allow distinct mechanical actions (i.e., simple mixing, conveying, or grinding) to occur. The reagent feed rate, screw, and barrel lengths and screw-rotating speed can be adjusted to optimize reaction conditions. As the mechanochemical reaction occurs in a continuous process, SE can be considered a solid-state equivalent to solution-based flow reactors [24]. Extrusion was first patented by Joseph Bramah in the late 18^th^ century for the manufacture of lead pipes [25]. Since then, it has been employed in several industrial applications, including food, polymer, and pharmaceutical manufacturing.

A typical extrusion line consists of a feeder (volumetric or gravitational) and a heatable barrel containing either one (SSE) or two (TSE) screws that convey the material along the inner barrel, where it is subjected to shearing and mixing forces before exiting. Optionally, a die can be attached to the exit to shape the material [26]. The extruder can easily be integrated into a continuous manufacturing process as either one component of a processing line or as the entire line itself. Several extrusion parameters can be tuned to optimize the process, including screw speed, screw profile, feed rate, and temperature. Furthermore, various output parameters can be monitored both during and after the extrusion process, and these parameters include throughput rate, which is the amount of product (kg) produced per hour, and the residence time, which is the time required for the material to pass through the extruder (typically from a few seconds to a few minutes, depending on the screw speed). The space-time yield (STY), i.e., the amount of product manufactured per unit reactor volume per day (kg m^−3^ day^−1^), is an engineering parameter that can be readily calculated as a measure of the overall efficiency of the process. Another important processing parameter that can be monitored is the torque, which represents the force applied to rotate the screws. Additionally, extrusion can be combined with on-line monitoring, via Raman or infrared spectroscopy, for example, which can provide vital information on the chemical changes occurring inside the extruder [27].

Extrusion processes are scalable up to tons of product per hour due to extensive engineering research. The main applications of extrusion can be found in polymer manufacturing, especially in the dispersion of materials into polymers [28]. Reactive extrusion (REX) [29] is used to synthesize polymers and to carry out post synthetic polymer modification (e.g., functionalization of polymer chains) via organic transformations that alter the properties of materials [30]. Moreover, these modifications can be achieved via four different REX processes: (i) grafting reactions; (ii) functionalization; (iii) controlled degradation; (vi) blending and reactive blending [31]. Besides polymer synthesis and processing, REX has recently been exploited for many other industrial applications, such as food processing [32,33], biomass conversion [11], pharmaceutical manufacturing [34], synthetic chemistry, and MOF synthesis [7,35,36]. Extrusion has also been widely explored as a means of intensifying pharmaceutical processes, such as drug formulation and their incorporation into drug-delivery systems [37]. However, it has not been extensively applied to the synthesis of active pharmaceutical ingredients (APIs). Some research, performed over the last decade, has demonstrated the preparation of co-crystals [38] via hot melt extrusion (HME) and liquid-assisted extrusion (LAE), while the first TSE-assisted synthesis of hydantoin-based APIs, namely dantrolene and nitrofurantoin, was only reported in 2020 by Crawford et al. [36].

In this context, this work highlights recent developments in the better-known applications of REX, starting from polymer processing and biomass conversion and finishing with catalyst preparation and oxidation reactions, and explores different case studies as a proof of concept for the potentiality of continuous mechanochemical transformations. In particular, two types of solvent-free oxidations were reported, exploiting Oxone^®^ as solid oxidant. The two benchmark organic reagents were drawn, aiming to two opposite goals: the oxidative degradation of dibenzothiophene, one of the sulfur contaminants from the oil industry, into commercial derivatives (fuels and waxes) and, on the other hand, the valorization of a lignin-like methoxylated aromatic substrate from biomass. Currently, the production of functional polymers has become a promising field of research. Herein we present the enrichment of the antioxidant or catalytic activity of new poly(ethylene-co-acrylic acid) (PEAA) blends obtained by co-extrusion with a β-cyclodextrin (CD) complex with polyphenols or metal ions, respectively. Finally, a fast solvent-free delignification of a lignocellulosic biomass was carried out in presence of NaOH, proving the efficiency of reactive extrusion in the solid-phase biomass pretreatment.

## 2. Results and Discussion

### 2.1. Single Screw Reactive Extruder (SSRE): Organic Synthesis Applications

A single screw reactive extruder (SSRE, see Figure S1 and Table S1 of the Supporting information) was first exploited for organic synthetic purposes in two solvent-free oxidative processes: the oxidation of lignin-like methoxylated aromatics [4], and the oxidation of organosulfur compounds [39]. Thanks to its high stability and efficiency, Oxone^®^ (2 KHSO_5_, KHSO_4_, and K_2_SO_4_) was selected as the oxidant agent for this purpose. It is a commercially available solid peroxygen for green, efficient non-chlorine oxidation reactions. Moreover, its degradation products are generally recognized as safe. Oxone^®^ has recently found many applications in the oxidation of alcohols [40], amines [41], and ketones [42], as well as in alkene epoxidation [43] and C–H bond oxidation processes [44].

**Scheme 1:**
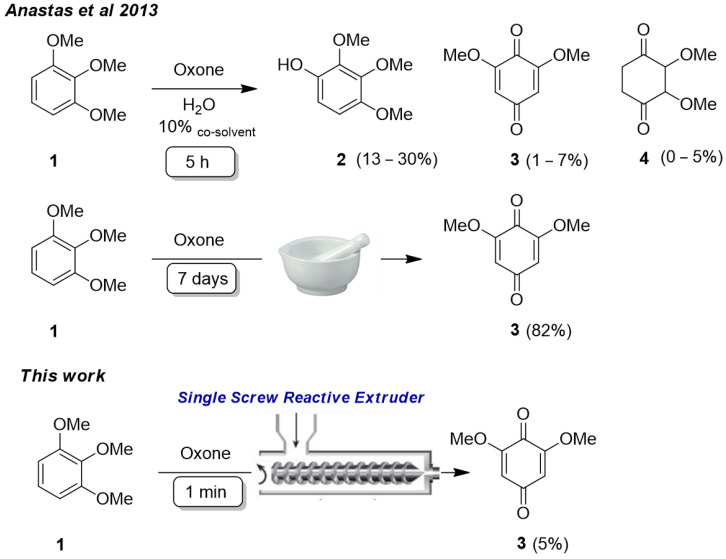
Oxidative processes of lignin-like monomers.

In 2013, Anastas and co-workers reported the mechanochemical oxidation of lignin-like methoxylated aromatic substrates **1** (Scheme 1) using Oxone^®^ [45]. When this reaction was carried out in aqueous solution, the major product was 2,3,4-trimethoxyphenol **2**, with several other side-products being observed. By contrast, quinone **3** was the only product formed under mechanochemical conditions. Seven days were required when this transformation was performed using a rock tumbler, while, in this study, fast oxidation was achieved by performing the reaction in a SSRE. Indeed, the methoxylated aromatic compound **1** was extruded together with Oxone^®^ in a molar ratio of (1:1) using SSRE at 90 °C (40 rpm). After only 1 min, 5% quinone **3** was the only product detected. This result is very promising as it paves the way for timesaving continuous oxidation reactions of lignin derivatives using SSRE (Figure S2).

Petroleum and paraffin contain a large variety of sulfur compounds (thiols, sulfides, disulfides, thiophenes, benzo, and dibenzothiophenes), which generate SO_2_ and airborne particulate emissions during combustion. Oxidative desulphurization (ODS) is attracting significant research interest, mainly as a means of treating light oils and paraffins, because of its ease of use and high efficiency [39].

**Scheme 2:**
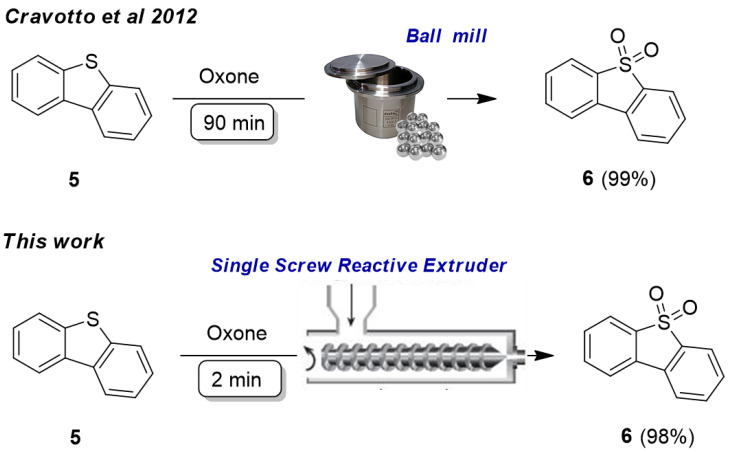
Mechanochemical oxidation of dibenzothiophene.

We herein report the selective solventless oxidation of dibenzothiophene (5) to sulfone **6** under reactive extrusion as a potential solution to the need for greener and more sustainable oxidative desulphurization methods (Scheme 2). Dibenzothiophene and Oxone^®^ were extruded at 90/100/110 °C in a molar ratio of 1:2, and 40% sulfone **6** was detected in only 1 min at 40 rpm. Using this encouraging achievement as a base, the same reaction was performed with double the extrusion residence time, and the yield increased to 98%.

This achievement may pave the way for the pilot-scale solventless oxidative desulphurization of paraffins, whose principal contaminant for removal is organic sulfur compounds.

### 2.2. SSRE: Polymer Applications

#### 2.2.1. Blended Polymer with Polyphenols Extracted from Residual Agri-Food Waste 

Antioxidants are widely known to be natural components of healthy foods and drinks, as well as additives to commercial polymer materials to prevent their degradation. Recent years have seen increasing interest in enhancing the antioxidant functionality of newly developed polymer materials and coatings [31,46].

We therefore focus this case study on the use of polyphenols as one of the most abundant classes of antioxidants (with over 8000 phenolic structures identified in plants), and one that can boast of powerful antioxidant activity, with the aim of bringing together the diverse areas of food science, analytical chemistry, and materials science, in the application of polyphenol antioxidant activity in polymer matrices. Different polyphenol−polymer interaction strengths can influence the release of polyphenols into aqueous and nonpolar media and can alter the antioxidant activity and physical properties of polyphenol-containing materials. Polyphenol-containing polymer materials can therefore be grouped into three main categories: (i) migratory materials; (ii) covalently bound non-migratory materials; and (iii) materials assembled via hydrogen bonding or electrostatic interactions that are capable of tenable antioxidant release in response to environmental conditions [46].

In this work, a polyphenol-poly(ethylene-co-acrylic acid) (PEAA) polymer blend was prepared in a SSRE. The synthetic PEAA polymer is endowed with good mechanical properties and hydrophilic functional groups [47].

PEAA thus has, in principle, the required features to yield mechanically strong blends that contain chemically compatible ingredients. Furthermore, its melting point is 108 °C, making the processing temperature lower than that of other synthetic polymers. This feature is crucial in this case, as higher temperatures could prompt the degradation of polyphenols, which are added as natural additives when forming the blended polymers via extrusion. Moreover, the presence of carboxylic groups, from the co-acrylic acid fraction, may favor the interaction between the polyphenols and the polymer matrix during extrusion.

**Figure 1:**
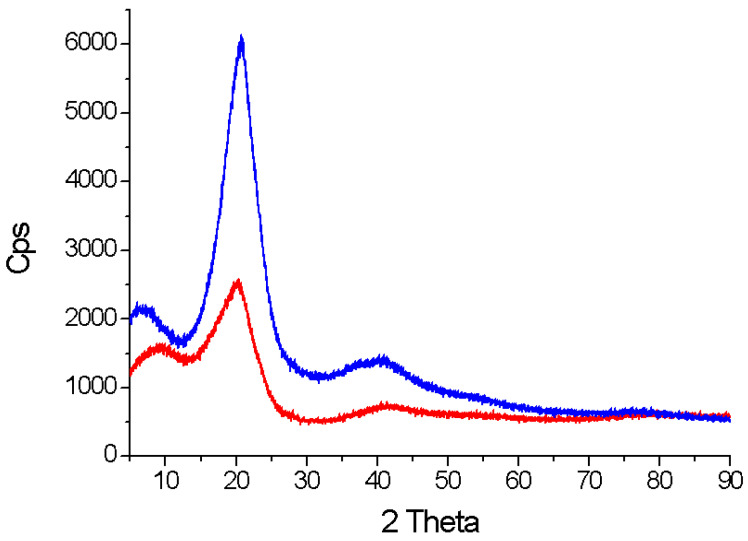
XRD patterns of PEAA before (red line) and after (blue line) extrusion at a temperature gradient of 90/100/110 °C.

The behavior of the PEAA polymer was monitored after extrusion. As can be seen in Figure 1, a comparison of the XRD patterns of pristine PEAA (red line) and PEAA after extrusion (blue line) at a temperature gradient of 90/100/110 °C shows that there is an increase in the intensity of the peaks at 2θ = 20.7° (most intense) and 2θ = 40.6° (less intense and broad), which is most likely due to an enhancement of crystallinity induced by the extrusion process.

The blend was obtained starting from PEAA (248 kDa molecular weight) and a polyphenol extract that was obtained from chestnut peels (CPPE) under microwave (MW)-assisted subcritical conditions (220 °C, 30 min) [48]. The CPPE, which displays antioxidant activity with an EC50 value of 0.14 mg/mL, was then introduced into a β-cyclodextrin (β-CD) matrix to enhance its handling during the extrusion process and to preserve its antioxidant activity.

β-CDs are cyclic oligosaccharides that are produced via the enzymatic degradation of starch and have a truncated cone-shaped structure with a hydrophilic outer surface and a lipophilic inner cavity [49]. These physicochemical properties allow them to capture guest molecules in their cavities and preserve them against degradation. With these properties in mind, a β-CD/CPPE inclusion complex with a host–guest ratio of 1:0.5 was prepared via co-precipitation before extrusion.

**Figure 2:**
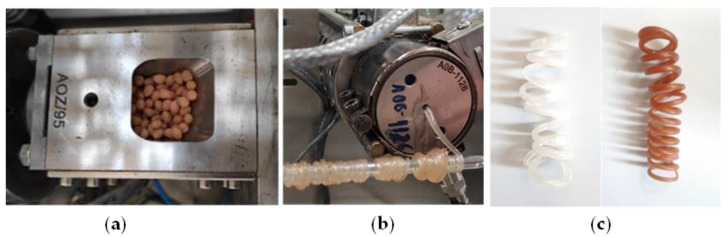
Main steps of blend preparation: (**a**) PEAA beads coated with β-CD/CPPE before extrusion, (**b**) extrusion of the β-*CD/CPPE-PEAA blend*, and (**c**) the obtained blended PEAA: β-*CD-PEAA* (**left**) and β-*CD/CPPE-PEAA* (**right**) for comparison.

This β-CD/CPPE complex (10% wt. loading) was then used in the PEAA blend, and extrusion was performed at 90–100–110 °C in a SSRE that was equipped with three differential heating units and that worked at 40 rpm for 1 min. PEAA blends were also extruded with CPPE alone for the sake of comparison. Figure 2 reports images of the PEAA beads coated with β-CD/CPPE before extrusion (Figure 2a), the extrusion of the β-CD-PEAA CPPE blend (Figure 2b) and the blended β-*CD-PEAA* (left) and β-*CD/CPPE-PEAA* (right) (Figure 2c).

The extrusion was performed at 90–100–110 °C in a SSRE that was equipped with three differential heating units and that worked at 40 rpm., and the pale brown polymer was obtained in only 1 min (Figure 2c).

**Figure 3:**
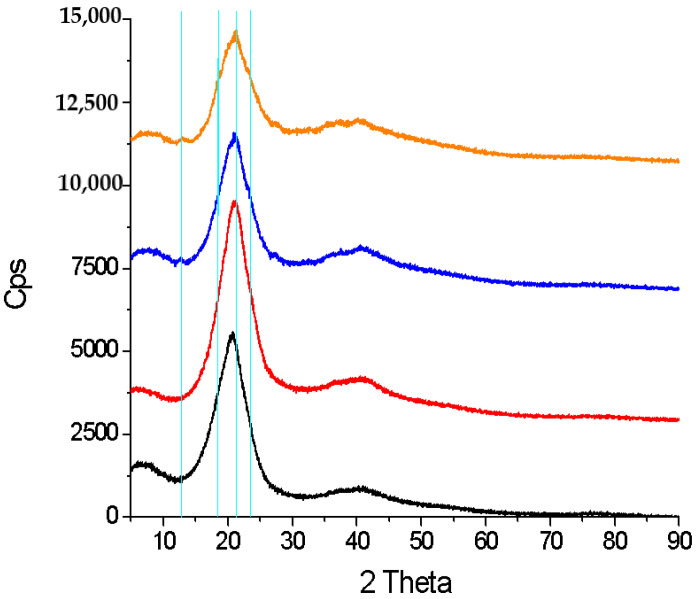
XRD patterns of extruded bare PEAA (black line), PEAA with the addition of CPPE (red line), PEAA with the addition of β-CD (blue line), and PEAA with the addition of the β-CD/CPPE complex (10% wt. loading) (orange line) at a temperature gradient of 90/100/110 °C. Cyan lines are eye guidelines to highlight peak positions.

XRD characterization was carried out on the obtained blends and revealed that the addition of bare CPPE did not modify PEAA crystallinity, with only a slight shift in the main peak toward higher angles being observed, which indicates that there was a small variation in the cell parameter, as shown in Figure 3 (red line vs. black line).

New peaks at 2 Theta, equal to 12.9°, 18.6°, 21.3°, and 23.3°, were observed in the XRD pattern upon the extrusion of PEAA in the presence of β-CD (Figure 3, blue line) and were also present in the pattern of the complex (orange line). In particular, the XRD pattern of the β-CD/CPPE complex (10% wt. loading, orange line) displays similar features to the those of PEAA with the addition of CPPE (red line) and the PEAA that was coextruded with β-CD (blue line); the shift of the main peak at 2θ = 20.7° and the presence of new β-CD-related peaks. These results confirm that the synthesis of an ordered blended material occurred and that the β-CD/CPPE complex moieties are regularly placed within the chains of the PEAA polymeric matrix.

**Figure 4:**
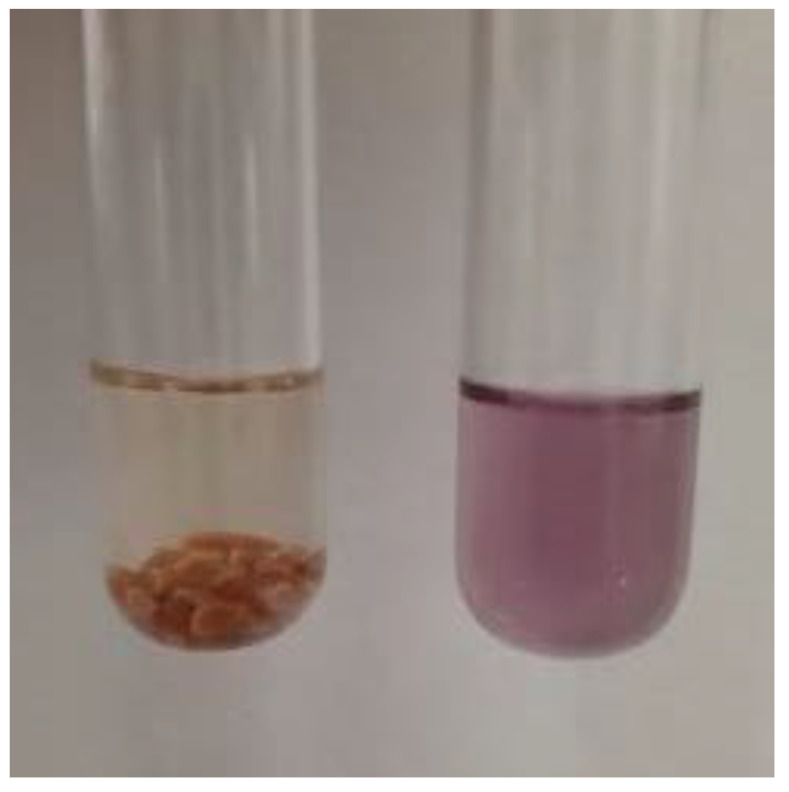
DPPH test: β-CD/CPPE-PEAA (**left**) and reference (**right**).

The antioxidant activity of the newly prepared blended polymer was then evaluated using its DPPH radical scavenging activity. The blended β-CD/CPPE-PEAA was left in contact with MeOH and the DPPH solution under stirring for 20 min at RT, and was protected from light (Figure 4). The recovered solution was then analyzed for its radical scavenging activity (DPPH inhibition essay, Figure S4) and the results show that the extruded blend still has a promising EC50 value, as reported in Table 1.

**Figure 5:**
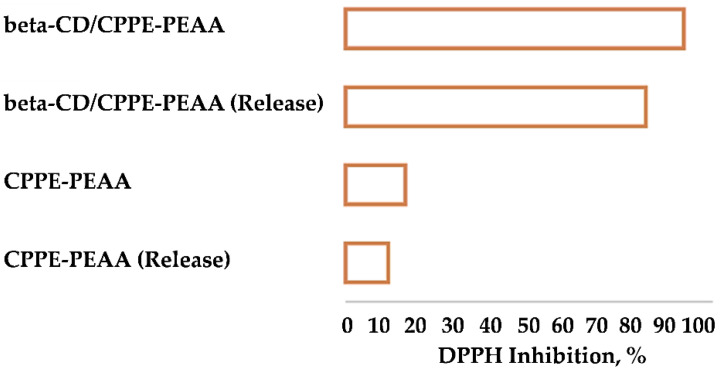
DPPH inhibition of blended polymers before and after release tests. The DPPH percentage inhibition was normalized to PEAA weight (40 mg).

To better understand the interactions that occur within the newly extruded polymer, release tests were performed by separately stirring 40 mg of β-CD/CPPE-PEAA and CPPE-PEAA into a MeOH solution (1 mL) for 20 min. The resulting solutions were then left in contact with the DPPH solution for a further 20 min in the dark in order to measure inhibition activity (Figure 5).

Results shed light on the possible nature of the different interactions that occur in the blended polymers. It is likely that stronger interactions occurred, in CPPE-PEAA, between the hydrophilic functionalities of PEAA and the extract, and that these interactions shield its antioxidant properties and prevent the release of polyphenols. On the other hand, in β-CD/CPPE-PEAA, hydrophilic interactions occurred between PEAA and the outer groups of β-CDs. These interactions enabled the proper inclusion of CD-CPPE complexes into the polymer framework, thus preventing the degradation of polyphenols included in β-CD during the extrusion. Indeed, when treated with MeOH, the polyphenols were released from the β-CD/CPPE-PEAA, still showing antioxidant activity.

These results therefore hold great promise for the synthesis of blended active polymers by reactive extrusion.

#### 2.2.2. SSRE Preparation of Palladium Catalytic Polymer

β-CD-based polymers have found many different applications over the last decade and can be synthesized using mechanochemistry [50].

**Figure 6:**
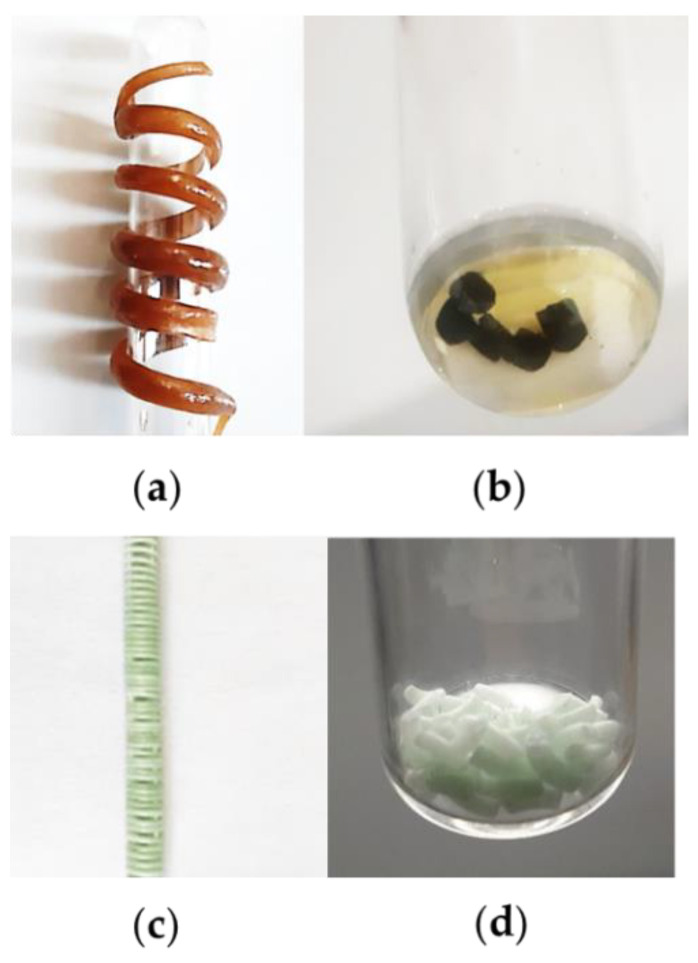
Pd/βCD-PEAA polymer (**a**) before and (**b**) after MW-assisted nitrobenzene hydrogenation and Cu/βCD-PEAA polymer (**c**) before and (**d**) after catalytic tests followed by filtration.

We have recently developed a reusable heterogeneous catalytic system in which Pd is embedded into a cross-linked β-CD matrix (a Pd/CβCAT system) [51,52], and that is obtained after the reticulation of β-CD with hexamethylene diisocyanate (HDI) in the presence of a Pd(II) salt solution [53]. On the base of these studies, we herein propose a method for the preparation of a new catalytic polymer that proceeds using extrusion and an inclusion complex between β-CD and Pd(AcO)_2_ (2.5% wt. Pd) (hereafter denoted the Pd/βCD complex). PEAA was extruded with the Pd/βCD complex (10% wt.) at 90–100–110 °C and 40 rpm, yielding a dark-brown hybrid polymer (Figure 6a).

The catalytic activity of this Pd/βCD-PEAA polymer was evaluated in the nitrobenzene (**7**) hydrogenation, which was selected as a benchmark reaction, and the results are summarized in Table 2. In particular, the amine **8** was obtained quantitatively when the reactions were performed under H_2_ pressure (10 bar) at room temperature using solvents that ranged from EtOH to toluene (Table 2, entries 1–3) in the presence of 50 mg of catalyst (some small pieces of the extruded Pd/βCD-PEAA polymeric material were cut and added to the reaction vial). Moreover, the MW technology was applied with the aim of reducing reaction time, and good results were achieved in only 30 min of irradiation (Table 2, entries 5 and 9).

Interestingly, the color of the Pd/βCD-PEAA polymer changed from dark brown into black after the reaction (Figure 6b), indicating that a modification of the Pd active phase occurred during nitrobenzene hydrogenation.

**Figure 7:**
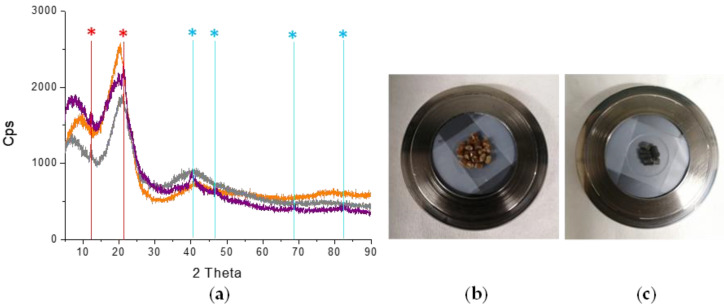
XRD patterns (**a**) and images of the Pd/βCD-PEAA polymer (Pd/βCD complex 10% wt. loading) mounted on the XRD spinning sample holder before (grey line and (**b**)) and after reaction (purple line and (**c**)). The XRD pattern of the bare extruded PEAA is also reported for the sake of comparison (orange line). The catalyst was synthesized using a temperature gradient 90/100/110 °C.

The reason of this change was investigated; the XRD analyses, carried out on the Pd/βCD-PEAA polymer before (grey line) and after (purple line) nitrobenzene hydrogenation, are reported in Figure 7. Based on the results from Figure 3, the peaks observed in the patterns at 12.3° and 21.2° (indicated by red asterisks) are related to the presence of ordered βCD moieties within the PEAA polymeric matrix, with the slightly different positions presumably being caused by interactions with palladium. Interestingly, metallic Pd is present in the cubic form (JCPDS file number 00-001-1201; cyan asterisks) after the reaction, as indicated by the new peaks at 2θ equal to 40.36° (corresponding to the 111 plane), 46.7° (200), 68.18° (220), and 82.30° (311) and by the change in the color of the Pd/®CD polymer from brown (Figure 6b) to dark grey (Figure 6b,c). It can therefore be stated that the Pd sites of the Pd/βCD inclusion complex between β-CD and Pd(AcO)_2_ (2.5% Pd wt.) underwent reduction under the reaction conditions and efficiently catalyzed nitrobenzene hydrogenation. Moreover, according to the observed full width at half maximum (FWHM), the peaks that are related to the metallic phase are broad. Despite the low intensity, a rough estimation of the size of the crystallites was performed using the Scherrer equation and a crystallite size of 2.7 nm was obtained, which confirms a high dispersion of the Pd active phase. 

FESEM measurements carried out on the Pd/βCD-PEAA catalyst before and after MW-assisted nitrobenzene hydrogenation revealed a modification of the surface morphology of the catalyst, which appears rougher and irregular after reaction (see Figure S5). However, the EDS analysis performed on different regions of the used catalyst did not put in evidence any Pd-rich region, indicating a high metal dispersion within the polymer (data not shown).

The results of the catalytic tests and the characterization indicate that: (i) the SSRE-based preparation delivers an efficient Pd/βCD-PEAA polymeric catalyst; (ii) the active Pd sites are readily reduced under the reaction conditions and are available for the MW-assisted hydrogenation of the nitrobenzene group; and (iii) βCDs play a role in Pd dispersion.

In addition, the catalytic results obtained in the benchmark reaction were compared to those obtained in the presence of another polymeric catalyst, which was prepared by following the same synthetic approach. In this case, CuSO_4_ was used as metal precursor to form the inclusion complex with β-CD (10% wt.) and then extruded with PEAA, obtaining a pale green material with 2.2% wt. Cu loading (Figure 6c). Differently from what observed for the Pd/βCD-PEAA catalyst, the Cu/βCD-PEAA material did not change its color at all after MW-assisted nitrobenzene hydrogenation (Figure 6d) according to the complete lack of activity reported in Table S2, using ethanol as the solvent. These results put in evidence that the Cu^2+^ sites are not available for the catalysis because they are likely bonded to βCD and strongly embedded in the PEAA matrix. Indeed, they are not reduced under reaction conditions neither by increasing the temperature up to 80 °C and 25 bar H_2_ pressure, nor by prolonging the reaction time up to 18 h. In the field of immobilized catalysts that operate under mild conditions at low catalyst loadings, Doherty et al. have recently described highly efficient reduction of nitroarenes catalyzed by Pd nanoparticles stabilized by ionic liquids immobilized on a phosphine-decorated PEG polymer [54]. Although this strategy has been successfully applied, major issues will hamper its implementation, owing to a complex and time-consuming catalyst preparation and the use of ligands. Conversely, Pd/βCD-PEAA catalyst described in this work is prepared by a simple and solvent-free SSRE procedure, affording high reaction yields under mild conditions.

### 2.3. SSRE Biomass Delignification

Profitable energy generation from alternative sources is essential to overcoming the need for fossil fuels, dwindling sources of petroleum, the rising price of oil and global warming. Biomass is a cheap and renewable energy source that is attracting ever more attention because of its high accessibility. Agricultural residues are now considered a cheap and sustainable energy source, with wheat straw being one of the abundantly available agricultural residues on a global level and Asia accounting for 90% of its annual generation worldwide. Cellulose, hemicellulose, and lignin are the main essential components of wheat straw and make it a good feedstock for bioethanol recovery. However, the presence of almost 19% lignin means that it is recalcitrant to direct enzymatic hydrolysis and further fermentation to bioethanol [55]. While a range of physical, chemical, and mechanical treatments, biopretreatments, and combined treatments have recently been reported in the literature [56], mechanical treatment is preferable because of its mild and almost solventless conditions and energy savings. Due to these favorable features, an ever-growing number of research studies have been published on the effectiveness of extrusion in producing fermentable sugars from a wide range of lignocellulosic biomasses. The results vary depending on the pretreatment conditions of the biomass, but, in all cases, extruded materials show higher sugar-production yields than the raw materials [57].

In the present study, wheat-straw biomass was subjected to mechanically induced delignification by reactive extrusion under basic conditions. In particular, two main approaches were chosen for wheat-straw delignification under SSRE: (i) the biomass was previously soaked in a NaOH aq. solution (10% wt. on dry biomass, s/l ratio: 1:20) overnight at room temperature and then extruded at either 130° or 190 °C for 1 min (40 rpm); and (ii) the biomass was extruded directly with NaOH beads at 190 °C for 1 min (40 rpm).

**Figure 8:**
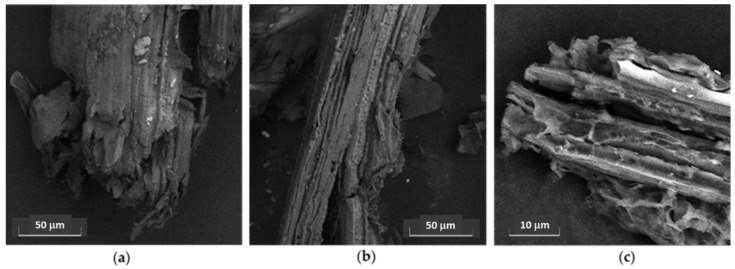
FESEM images collected on the as-received wheat-straw biomass before SSRE extrusion (**a**–**c**). FESEM images collected at 15 kV (**a**,**b**) and 5 kV (**c**) using a BSE detector. Instrumental magnification: 1500× (**a**,**b**) and 6000× (**c**).

Preliminary field emission scanning electron microscopy (FESEM) measurements were performed on the as-received wheat-straw biomass (see Figure 8), and it was observed that the untreated wheat-straw biomass is formed of compact and ordered bundles of small fibers with smooth and fairly intact surfaces [58].

Further analyses were subsequently carried out on the wheat-straw biomass that had undergone the two procedures reported above. The aim was to monitor the possible morphologic changes that occurred during extrusion.

**Figure 9:**
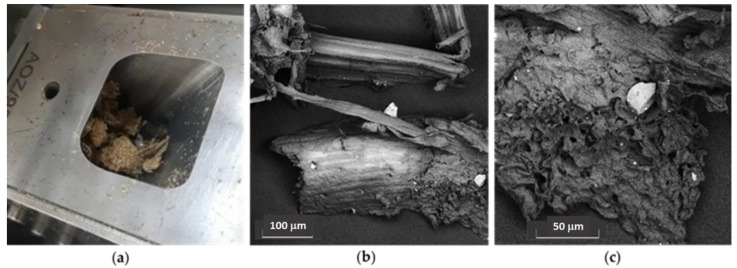
Digital image (**a**) and FESEM images of the wheat-straw biomass soaked with a NaOH solution (10% wt. on dry biomass) overnight at room temperature before (**a**) and after SSRE extrusion (**b**,**c**). FESEM images collected at 5 kV with a BSE detector. Instrumental magnification: 500× (**b**) and 1500× (**c**).

No apparent differences were found in the wheat-straw biomass after pretreatment with the NaOH solution (Figure 9a) or after SSRE extrusion at room temperature (Figure 9b), with the exception of some regions that were mainly located at the extremities of the fiber bundles, where some roughness was observed, which may indicate that fiber decomposition occurred via hydrolysis upon NaOH treatment (Figure 9c).

**Figure 10:**
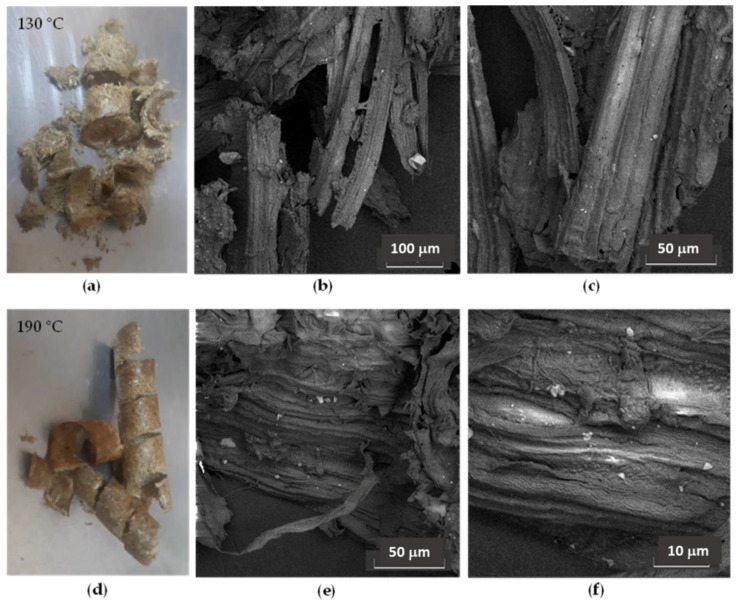
Digital images (**a**,**d**) and FESEM images of wheat-straw biomass that underwent soaking with a NaOH solution (10% wt. on dry biomass) overnight at room temperature before SSRE extrusion at 130 °C (**b**,**c**) and 190 °C (**e**,**f**). FESEM images collected at 5 kV with a BSE detector. Instrumental magnification: 600× (**b**), 1500× (**c**,**e**), and 6000× (**f**).

The fiber bundles became loose when the wheat-straw biomass was extruded at 130 °C (Figure 10a–c), and even split into long ribbons, which were exfoliated from the main bundles, while the surface appeared rougher (Figure 10d–f), indicating that the material was more disordered when treated at 190 °C.

**Figure 11:**
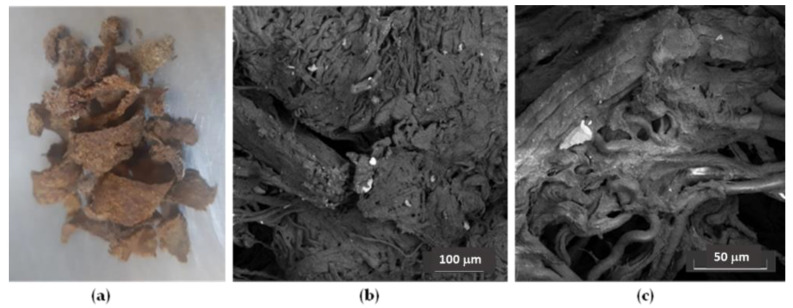
Digital image (**a**) and FESEM images of the wheat-straw biomass that was coextruded with NaOH beads at 190 °C for 1 min at 40 rpm (**b**,**c**). FESEM images collected at 5 kV with a BSE detector. Instrumental magnification: 500× (**b**) and 1500× (**c**).

The FESEM images of the wheat-straw biomass that were extruded with NaOH beads, at 190 °C for 1 min at 40 rpm without pretreatment, are shown in Figure 11. SSRE co-extrusion seriously compromised the fibrous morphology of the wheat-straw biomass, which appeared disintegrated and brownish (Figure 11a). Indeed, it is possible, even at low magnification, to see that the material is very disordered (Figure 11b), and these features are even more evident at higher magnification; the flat and fibrous bundles that were observed after extrusion at 130 °C and 190 °C became roundish and bent noodles without any preferential orientation (Figure 11c).

After extrusion in both conditions, the solid residue was characterized by NREL [59], to determine the residual lignin content. Starting from a 21.8% lignin content in the raw biomass, a higher lignin-removal of 20% on raw biomass (see Table S2) was achieved when the wheat straw was extruded directly at 190 °C (40 rpm) in the presence of NaOH beads, without pretreatment, whereas that value was 15% when wheat straw was pretreated overnight with NaOH and extruded at 190 °C. The direct and fast extrusion in presence of NaOH beads enabled better delignification, avoiding long time pretreatment with aqueous NaOH, thus preventing wastewater production (20 L/kg).

The resulting solid residue was thus enriched in polysaccharide fraction, for further saccharification purposes, paving the way to SSRE simple application for sustainable biomass delignification. Similar results were reported previously by Lauberte et al. [60] through an ultrasound-assisted process, exploiting NaOH solutions for longer treatment times (60 min instead of 1 min with SSRE).

## 3. Material and Methods

### 3.1. Materials

Polyethylene-co-acrylic acid, acrylic acid 20 mol % (PEAA), CAS number 9010-77-9, and all other reagents and solvents were supplied by Sigma Aldrich (Milan, Italy) and used as received.

Wheat straw was provided in milled form (0.2 mm) by Environmental System GmbH (Bremerhaven, Germany). The matrix was stored in a dry place until use. The wheat-straw consisted of 21.8% (*w*/*w*) total lignin and 8.5% (*w*/*w*) inorganic materials (ash) [61].

### 3.2. General Procedure for the Solvent-Free Oxidation of Organic Compounds

Solvent-free oxidation was conducted on two different substrates: dibenzothiophene and 1,2,3-trimethoxybenzene. For both compounds, Oxone^®^ (2 KHSO_5_, KHSO_4_, and K_2_SO_4_) was adopted as oxidizing agent. For the SSRE reaction, Oxone^®^ was mixed in a 1:2 and 1:1 molar ratio for dibenzothiophene and 1,2,3-trimethoxybenzene, respectively. Deionized water was used to suspend the substrates achieving a slurry, subsequently lyophilized. The achieved solid pads were crushed in chunks of approximately 1 × 1 cm, and then fed to the extruder kept at 40 rpm. ODS was conducted setting heating moduli at 90/100/110 °C, whilst for the methoxylated aromatic compound the system was maintained at 90 °C. The recovered solids were extracted in CHCl_3,_ and the solutions were analyzed by GC-MS (Agilent Technologies 6850 Network GC System equipped with a 5973 Network Mass Selective Detector, 7683B Automatic Sampler and a capillary column Mega 5MS with length 30 m, i.d. 0.25 mm and film thickness 0.25 μm, Mega S.r.l., Milan, Italy). Compounds quantifications were reported as GC–MS percentage areas.

### 3.3. Preparation of the β-CD/CPPE Inclusion Complex

The β-CD/CPPE inclusion complex was prepared at a molar ratio of 1:0.5. Once the β-CD was fully dissolved in 40 mL of 30% ethanol in a conical flask that was wrapped in an aluminum-foil cone, the chestnut peel polyphenol extract was added to its final concentration, and the solution was shaken at 25 °C for 8 h prior to rotary evaporation and placed in a refrigerator at 4 °C overnight. The sediment was obtained using vacuum filtration and washed with ethanol, then frozen at −80 °C for 24 h and freeze-dried in a freeze dryer for 12 h to give the resulting solid complexes.

### 3.4. MW-Assisted Nitrobenzene Hydrogenation Using the Pd/B-CD-PEAA Polymer

In a typical experiment, 50 mg of catalyst were added to 100 μL of nitrobenzene, together with 1 mL of the desired solvent (EtOH, cyclopentyl methyl ether (CPME), toluene, 2-methyltetrahydrofuran and i-PrOH). The mixture was placed in a borosilicate vessel and maintained under magnetic stirring (200 rpm) at the target temperature (60 °C or RT) for the required time, in a multimode MW reactor (SynthWAVE Milestone Srl, Bergamo, Italy). This system is capable of providing power up to 1500 W at a working frequency of 2.45 GHz. Before every run, the reactor was filled with 10 bars of O_2_. After the process, the reaction mixtures were filtered under vacuum to remove the catalyst, and 100 μL of the resulting solution was diluted with 500 μL of chloroform. Analyses were carried out on a GC–MS (Agilent Technologies 6850 Network GC System equipped with a 5973 Network Mass Selective Detector, 7683B Automatic Sampler and a capillary column Mega 5MS with length 30 m, i.d. 0.25 mm and film thickness 0.25 μm, Mega S.r.l., Italy). Compound quantifications were reported as GC-MS percentage areas.

### 3.5. Biomass Pretreatment

In a typical pretreatment, WS was soaked overnight in an alkaline solution (NaOH, 10% on dry biomass weight), respecting a 1:20 S/L ratio. The mixture was pressed in a TC press (Multigel Srl, Florence, Italy) to remove the liquid. The solid pads were shredded in chunks of approximately 1 × 1 cm, fed into the extruder kept at 40 rpm. Pretreatment temperature was screened in a range of 110 °C to 190 °C, keeping a difference of 10 °C between adjacent heating moduli (i.e., 110/120/130 °C and 170/180/190 °C). Concerning the solvent-free pretreatment protocol, chunks of un-soaked WS (dimension approximately 1 × 1 cm) were fed into the extruder (40 rpm, 170/180/190 °C). NaOH beads were introduced concurringly, with a WS/NaOH ratio of 3 *w*/*w*, to preserve an appropriate dispersion of the two solid phases.

After the extrusion, the pretreated WS was washed with water to remove NaOH and lignin. Solid fraction was recovered by centrifugation and freeze-dried (LyoQuest–85, Telstar, Spain). Treated and untreated WS were analyzed according to NREL protocol [59]. Ashes, extractives, carbohydrates, and lignin contents are reported in Table S3.

### 3.6. Methods

X-ray diffraction (XRD) patterns were collected using a PW3050/60 X’Pert PRO MPD diffractometer (PANalytical) (Malvern, UK) working in Bragg–Brentano geometry using, as the source, a high-powered ceramic tube, PW3373/10 LFF, equipped with a Cu anode (Cu K_α1_ radiation λ = 1.5406 Å) and a Ni filter to attenuate *K*_β_. Scattered photons were collected using a real time multiple strip (RTMS) X’celerator detector. Data were collected in the 5° ≤ 2θ ≤ 90° angular range with 0.02° 2θ steps. The XRD characterization of the materials was performed using the spinner experimental set-up, which has been proven effective for data acquisition (see Figure S3 of the Supporting information). The samples were examined in their as-received forms and placed in a spinning sample holder to avoid preferred orientations.

The Scherrer equation was applied to relate the size of the crystallites to the broadening of the peaks in the diffraction pattern; τ is the mean size of the crystalline domain, K represents a dimensionless shape factor, with a value of about 0.9, λ is the X-ray wavelength, in this case, λ = 1.5406 Å, β is the line broadening at half the maximum intensity (FWHM, expressed as radians of 2θ), and θ is the Bragg angle.

Field emission scanning electron microscopy (FESEM) measurements were carried out using a TESCAN S9000G FESEM 3010 microscope (30 kV) (TESCAN ORSAY HOLDING, a.s., Brno-Kohoutovice, Czech Republic), equipped with a high brightness Schottky emitter and Energy Dispersive X-ray Spectroscopy (EDS) (Oxford, Abingdon-on-Thames, UK) analysis thanks to a Ultim Max Silicon Drift Detector (SDD, Oxford, Abingdon-on-Thames, UK). The samples were deposited on a stub that was coated with a conducting adhesive and inserted into the chamber in a fully motorized procedure. The samples were observed in their as-prepared forms and no metallization was performed to avoid surface-morphology modification.

## 4. Conclusions

Besides the green advantages, there are a number of limitations and drawbacks to the scale-up to industrial production of mechanochemical synthesis, which has not yet been fully explored. The critical issue of temperature control in ball milling can be overcome using reactive extruders. Extrusion is commonly applied in the polymer, materials, and food industries, whereas it is almost unknown in fine-chemical production. Recent advances in reactive micro-extruders appear to have paved the way for their use in organic synthesis. We believe that this perspective article, which has critically summarized the progress made in the field of mechanochemical multicomponent chemistry, may be able to drive researchers to consider this technology as a viable approach to green process intensification in chemical transformations. Mechanochemical treatments performed using extrusion are capable of making a remarkable contribution to the scale-up of environmentally benign chemical syntheses, not least by removing the enormous impact of organic solvents.

## Figures and Tables

**Table 1:** Scavenging activity of extract and extruded blend.

Samples	EC50 (mg/mL)	Trolox eq. (µmol/g _EXT_)
CPPE	0.14	112.44
β-CD/CPPE-PEAA	0.48	32.80

**Table 2:** Pd/βCD-PEAA catalytic tests.

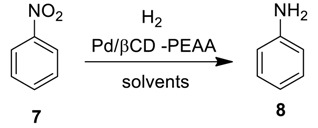					
Entry	Solvent	Time (min)	T (°C)	Yield (%) ^a^	Selectivity (%)
1	EtOH	18 h	RT	100	100
2	i-PrOH	18 h	RT	100	100
3	Toluene	18 h	RT	100	100
4 ^c^	EtOH	15	60	29.91	91.8
5 ^c^	EtOH	30	60	65.2	65.2 ^b^
6 ^c^	i-PrOH	15	60	10.0	100
7 ^c^	i-PrOH	30	60	22.1	99
8 ^c^	Toluene	15	60	10.3	100
9 ^c^	Toluene	30	60	88.3	98.9

^a^ GC yields; ^b^ by product formation. ^c^ MW-assisted hydrogenation. Reaction conditions: H_2_: 10 bar, stirring 200 rpm, 50 mg catalyst (2.2% wt. Pd on βCD-PEAA), 100 µL nitrobenzene, S/L 1:10 (1 mL solvent) (see Figures S6 and S7 for control tests).

## Data Availability

Not applicable.

## Supplementary Materials

The following are available online. Figure S1: Micro Extruder Ø12/24D (Gimac Int. srl); Table S1: Technical features of Micro-extruder; Figure S2: GC-MS chromatogram of lignin-derived methoxylated substrate **1**; Figure S3: XRD patterns and images of PEAA mounted on different XRD sample holders before (black and red lines) and after extrusion (orange and blue lines); Figure S4: DPPH inhibition test for β-CD/CPPE-PEAA. Probit regression, relative equation and EC50 value; Figure S5: FESEM images collected on the Pd/βCD-PEAA polymeric catalyst (a) before and (b) after MW-assisted nitrobenzene hydrogenation. FESEM images collected at 10 kV using a SE detector. Instrumental magnification: 1170× (a) and 1370× (b); Figure S6: Pd/βCD-PEAA polymer before SSRE; Figure S7: Pd/βCD-PEAA polymer after MW-assisted nitrobenzene hydrogenation: control experiments; Table S2: Cu/βCD-PEAA catalytic tests; Table S3: WS biomass characterization, NREL method [1].

## Sample Availability

Samples of the compounds are available from the corresponding author Cravotto, G.* at Department of Drug Science and Technology; giancarlo.cravotto@unito.it.
