# Supplementary material for: Mechanochemical Applications of Reactive Extrusion from Organic Synthesis to Catalytic and Active Materials
Source: Molecules. 2022 Jan 10;27(2):449. doi: 10.3390/molecules27020449 (PMC8779840; doi:10.3390/molecules27020449)
Supplement: Supplementary file 1 [file molecules-27-00449-s001.zip › molecules-1475139-supplementary.pdf]

Supporting information

Mechanochemical Applications of Reactive Extrusion from Organic Synthesis to Catalytic and Active Materials

Emanuela Calcio Gaudino [1], Giorgio Grillo [1], Maela Manzoli [1], Silvia Tabasso [2], Simone Maccagnan [3] and Giancarlo Cravotto [1,4,*]

[1] Department of Drug Science and Technology, University of Turin, Via P. Giuria 9, 10125 Turin, Italy; emanuela.calcio@unito.it (E.C.G.); giorgio.grillo@unito.it (G.G.); maela.manzoli@unito.it (M.M.)

[2] Department of Chemistry, University of Turin, Via P. Giuria 7, 10125 Turin, Italy; silvia.tabasso@unito.it

[3] GIMAC International srl., Via Roma 5, 21040 Castronno, Italy, simone.maccagnan@gimac.com

[4] World-Class Research Center, Sechenov First Moscow State Medical University, 8 Trubetskaya ul, 119991 Moscow, Russia

* Correspondence: giancarlo.cravotto@unito.it; Tel.: +39-011-670-7183

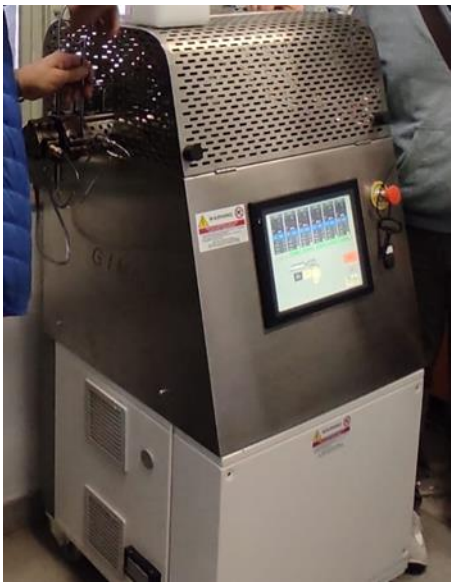

a

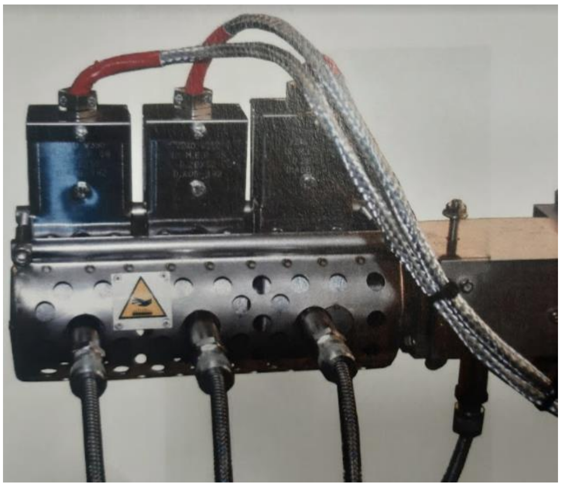

b

Figure S1. Micro Extruder Ø12/24D (Gimac Int. srl) a) Micro extruder Ø12/24D unit; b) Extrusion chamber equipped with three independent thermoregulation zones and their own corresponding thermocoup

The Micro Extruder Ø 12/24D (Figure S1) is made up of: 1) a B.O.F.S. type support base; 2) an engine (0.75 Kw, 250 Hz, 4.1 A, 230 V) equipped with a VSF adapter; 3) a feeding section with hopper 4) an extrusion chamber equipped with three independent thermoregulation zones and a single screw. The main technical features are reported in Table S1.

Table S1. Technical features of Micro-extruder

EXTRUDER Ø12/24D	Technical features
Screw diameter [mm]<br>Screw length [L/D]<br>Maximum Screw speed [RPM]	12<br>24<br>100
N° of barrel heating zones<br>Max. working temperature [°C]<br>Max. working pressure [Bar]	3<br>350<br>750
Hopper capacity [litres]	3
N° of cooling zones of the feeding zone<br>Type of cooling<br>Water consumption [m^3/h]	2<br>water<br>~0.1
Motor type<br>N° of revolution [RPM]<br>Power of motor fan [W]<br>Encoder type	asynchronous<br>2000<br>40<br>1024 imp/rpm
Reduction gear type<br>Reduction gear ratio [I:]	worm reduction<br>20
Total Installed power KW<br>Extruder weight Kgs	2.2<br>32

This work

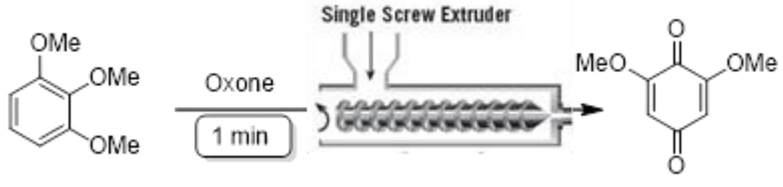

1   3 (5%)

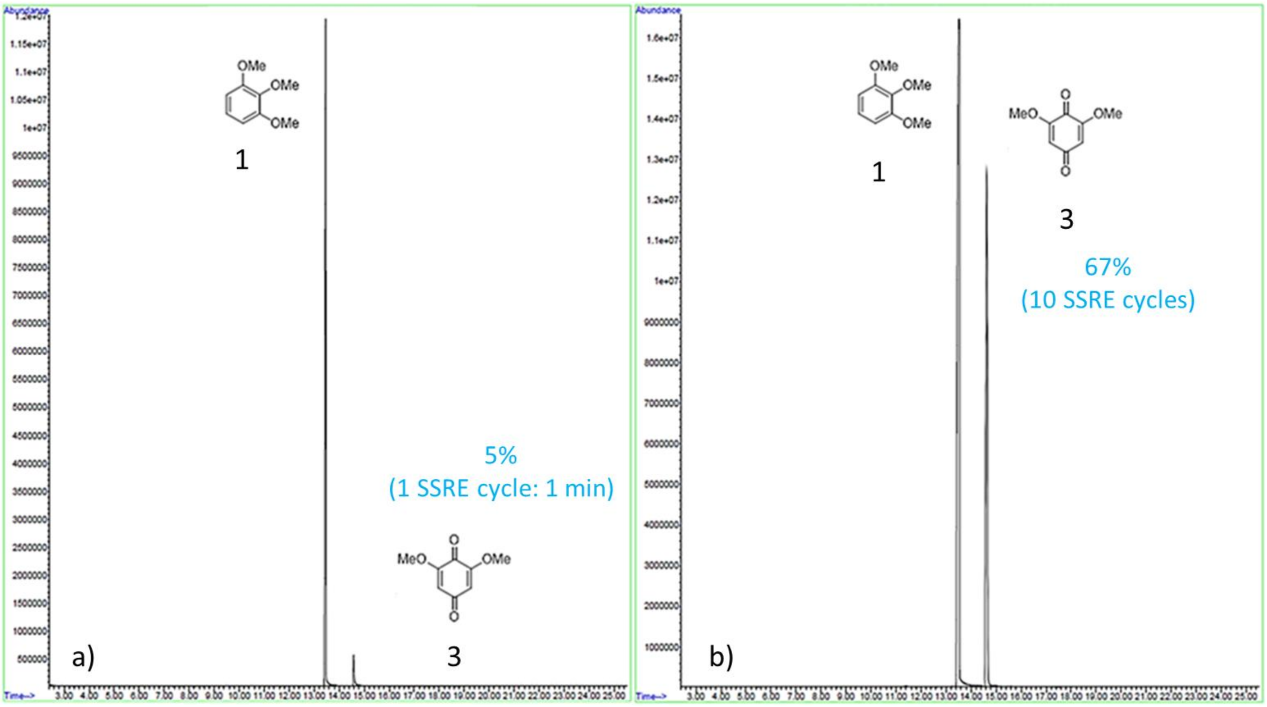

Figure S2. GC-MS chromatogram of lignin-derived methoxylated substrate 1. a) The GC-MS chromatogram of lignin derived methoxylated compound 1 oxidation b) the GC-MS chromatogram of a multiple extrusion was reported for sake of clarity to document the yield improvement in term of quinone 3.

SI-2 Single screw reactive extruder (SSRE): organic synthesis applications

The GC-MS chromatogram of lignin derived methoxylated compound 1 oxidation was reported in Figure S2a proving that no other side products were recorded for the SSRE oxidation in 1 min (Figure S2a).

In addition, the GC-MS chromatogram of a multiple extrusion was reported for sake of clarity to document the yield improvement in term of quinone **3** (until 67 % after 10 cycles of SSRE extrusion in the same conditions) (Figure S2b). However, the recycling also involves a certain handling trouble that could be avoided by stretching the **Micro Extruder Ø12/24D** screw unit.

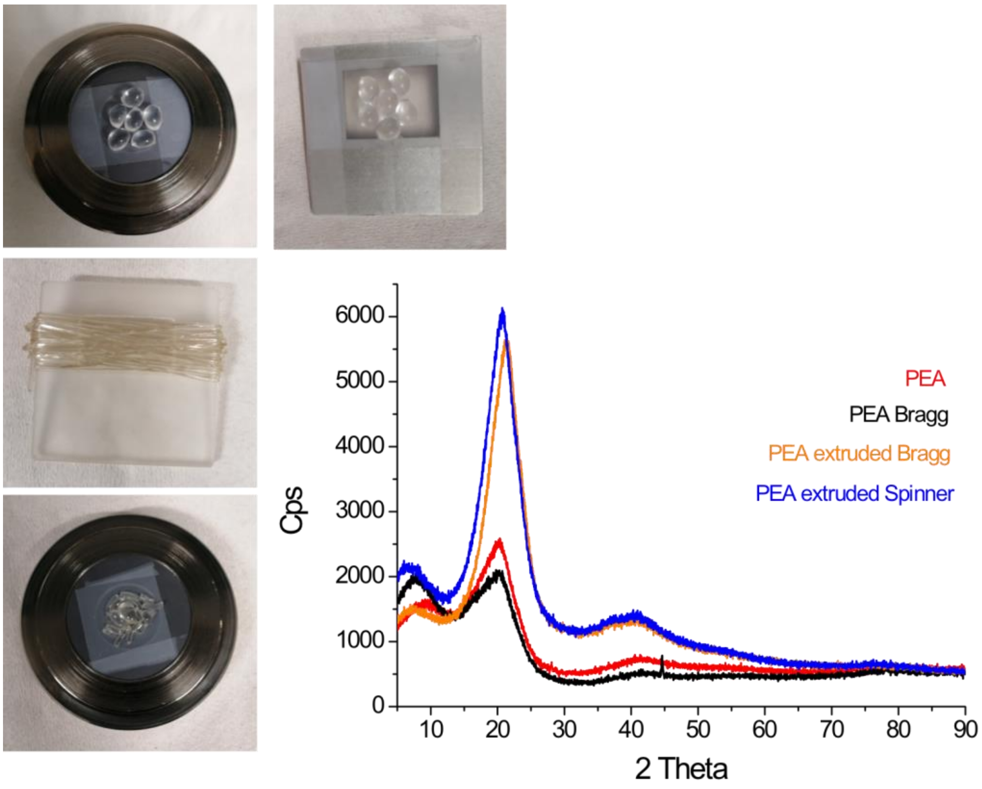

Figure S3. XRD patterns and images of PEAA mounted on different XRD sample holders before (black and red lines) and after extrusion (orange and blue lines).

SI-3 PEAA-CP-PE Blended polymer with polyphenols extracted from residual agri-food waste: chestnut peels

A polyphenol-poly(ethylene-co-acrylic acid) (PEAA) polymer was exploited to prepare the new PEAA- chestnut peels (CP) polyphenols extract (PE) blend using the Micro Extruder Ø 12/24D device.

These preliminary data put in evidence that XRD patterns can be collected by using the spinner experimental setup without any contribution from the adhesive employed to fix the samples.

Moreover, the antioxidant activity of newly prepared blended polymer β-cyclodextrin /CPPE-PEAA was then evaluated measuring its DPPH radical scavenging activity (Figure S3).

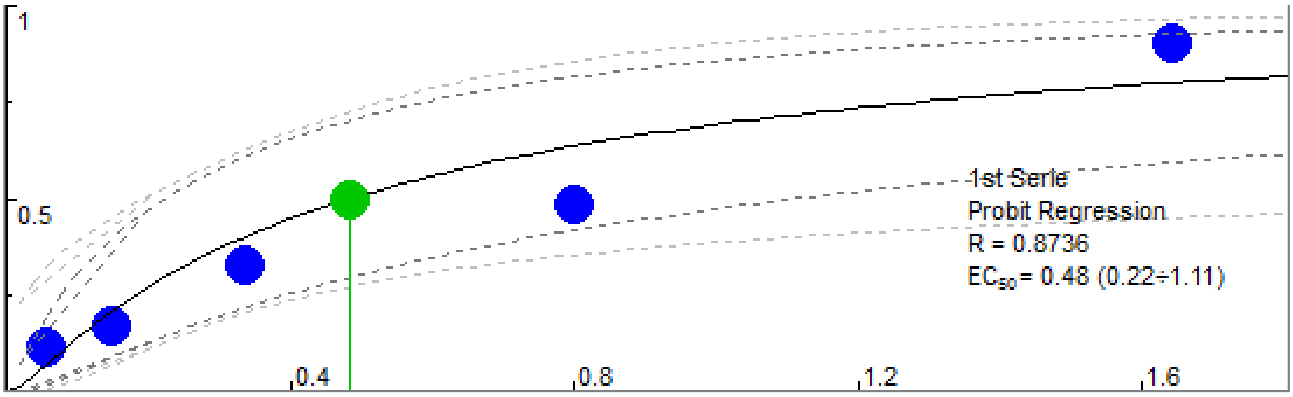

Figure S4. DPPH inhibition test for β-CD/CPPE-PEAA. Probit regression, relative equation and EC50 value.

SI-4 SSRE preparation of palladium catalytic polymer

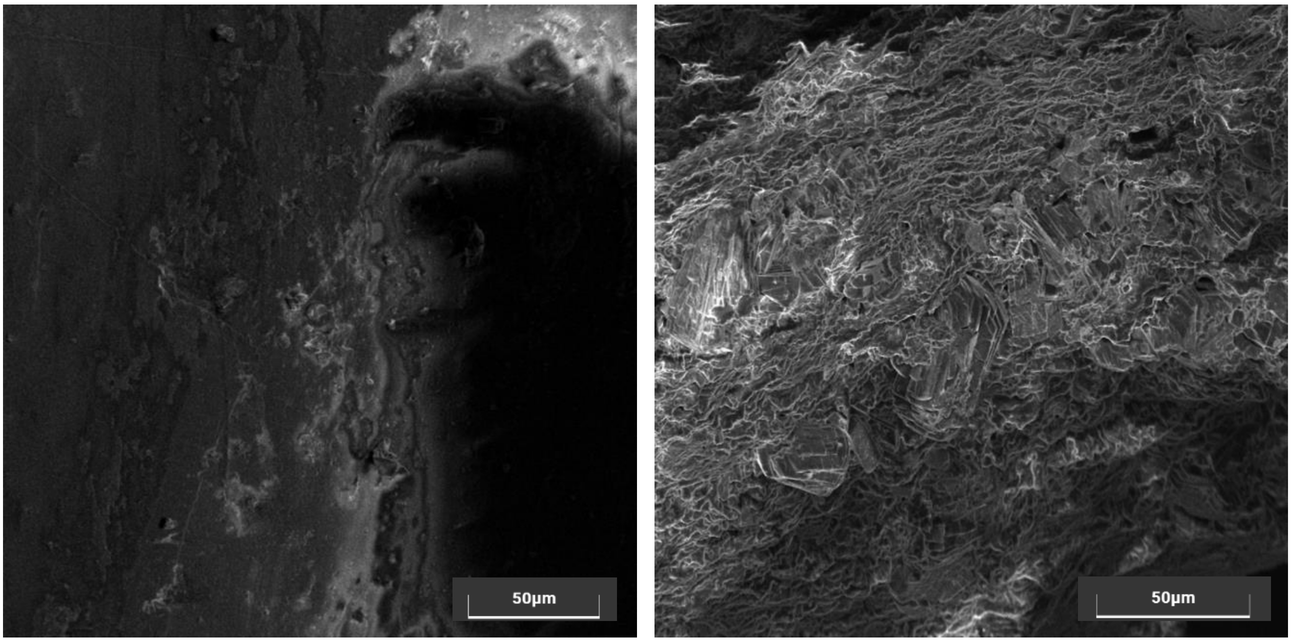

(**a**) (**b**)

Figure S5. FESEM images collected on the Pd/βCD-PEAA polymeric catalyst (a) before and (b) after MW-assisted nitrobenzene hydrogenation. FESEM images collected at 10 kV using a SE detector. Instrumental magnification: 1170× (a) and 1370× (b).

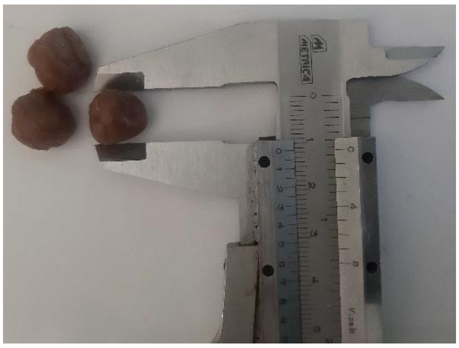

Figure S6. Pd/βCD-PEAA polymer before SSRE

The starting Pd/bCD complex plus PEAA before extrusion is not suitable for catalytic purposes, as the surface area is too low (see Figure S6).

An extruded Pd/PEAA polymer without β-CD addition as well as a Pd/β-CD catalyst without PEAA were used as reference catalysts to perform the nitro benzene reduction under MW conditions (60°C, 30 min) in toluene for sake of comparison.

Despite similar product yields were obtained, both the Pd/ βCD complex plus PEAA before extrusion and the Pd/PEAA polymer enabled a visible and remarkable Pd leaching (Figure S6), not recorded with the extruded Pd/β-CD-PEAA catalyst, making the latter a promising recyclable palladium catalyst.

**Pd/ CD complex**	**Pd// CD with PEAA polymer**	**Pd/βCD-PEAA polymer after extrusion**
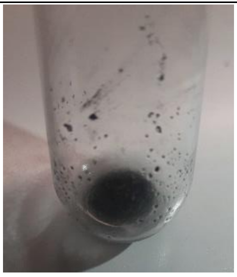	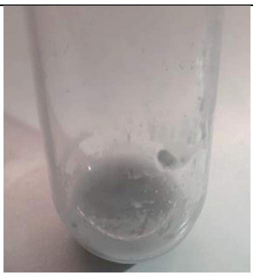	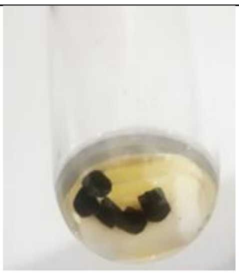
**a)**	**b)**	**c)**

Figure S7. Pd/βCD-PEAA polymer after MW-assisted nitrobenzene hydrogenation: control experiments

Table S2. Cu/βCD-PEAA catalytic tests.

Entry	Time	T°C	H_2 (bar)	Conversion (%)
1	30 min	60	10	no
2	30 min	80	10	no
3	18 hrs	RT	10	no
4	18 hrs	60	10	no
5	18 hrs	80	10	no
6	18 hrs	80	25	no

Reaction conditions: EtOH, stirring 200 rpm, 50 mg catalyst (2.2 wt% Cu on PEA), 100 μL nitrobenzene, S/L 1:10 (1 mL solvent).

Table S3. WS biomass characterization, NREL method. [1]

**Wheat straw (WS)**	Ash (%, DM)	Extractives (%, DM)	**Carbohydrates (%, DM)**	**Lignin (%, DM)**		
			TOT	Acid insol.	Acid sol.	TOT
**WS-raw**	7.5	0.7	70.0	20.6	1.2	21.8
**SSRE-WS1 130**	9.8	0.5	81.2	12.6	0.8	8.4
**SSRE-WS1 190**	9.3	0.3	83.6	5.6	1.2	6.8
**SSRE-WS2-190**	9.0	0.4	88.8	0.6	1.2	1.8

SSRE-WS1: wheat-straw biomass that underwent soaking with a NaOH solution (10 % wt. on dry biomass) overnight at room temperature before SSRE extrusion at 130°C and 190 °C.
SSRE-WS2: wheat-straw biomass that underwent SSRE extrusion at 190 °C with NaOH beads.

Reference

1. Genevini, P.; Adani, F.; Villa, C.; Rice hull degradation by co-composting with dairy cattle slurry. *Soil Sci. Plant. Nutr.* **1997**, *43*, 135–147. https://doi.org/10.1080/00380768.1997.10414722
